# Increased Risk of Suicide among Cancer Survivors Who Developed a Second Malignant Neoplasm

**DOI:** 10.1155/2022/2066133

**Published:** 2022-01-10

**Authors:** Huazhen Yang, Yuanyuan Qu, Yanan Shang, Chengshi Wang, Junren Wang, Donghao Lu, Huan Song

**Affiliations:** ^1^West China Biomedical Big Data Center, West China Hospital, Sichuan University, Chengdu, China; ^2^Med-X Center for Informatics, Sichuan University, Chengdu, China; ^3^Sichuan Cancer Hospital & Institute, Sichuan Cancer Center, School of Medicine, University of Electronic Science and Technology of China, Chengdu, China; ^4^Laboratory of Molecular Diagnosis of Cancer, Clinical Research Center for Breast, West China Hospital, Sichuan University, Chengdu, China; ^5^Institute of Environmental Medicine, Karolinska Institute, Stockholm, Sweden; ^6^Department of Epidemiology, Harvard T. H. Chan School of Public Health, Boston, MA, USA; ^7^Center of Public Health Sciences, Faculty of Medicine, University of Iceland, Reykjavík, Iceland

## Abstract

**Background:**

Cancer diagnosis entails substantial psychological distress and is associated with dramatically increased risks of suicidal behaviors. However, little is known about the suicide risk among cancer survivors who developed a second malignant neoplasm (SMN).

**Methods:**

Using the Surveillance, Epidemiology, and End Results database, we conducted a population-based cohort study involving 7,824,709 patients with first malignant neoplasm (FMN). We measured the hazard ratios (HRs) of suicide death after receiving a SMN diagnosis using Cox proportional hazard models, as compared with patients with FMN. The comparison with the US population was achieved by calculating standardized mortality ratios (SMRs).

**Results:**

Totally 685,727 FMN patients received a diagnosis of SMN during follow-up, and we in total identified 10,930 and 937 suicide deaths among FMN and SMN patients, respectively. The HR of suicide deaths was 1.23 (95% confidence interval (CI), 1.14–1.31) after a SMN diagnosis, compared with FMN patients, after adjusting for sociodemographic factors, tumor characteristics, and cancer treatment. As compared with the general population, while both SMN and FMN patients suffered an increased risk of suicide deaths, the excess risk was higher among SMN patients than FMN patients (age-, sex-, and calendar-year-adjusted SMR 1.65 (95% CI 1.54–1.75) vs. 1.29 (95% CI 1.26–1.31); *P*_difference_ < 0.0001). Notably, across different time periods, we observed the greatest risk elevation during the first 3 months after a cancer diagnosis.

**Conclusions:**

Compared with either patients with FMN or the general population, cancer survivors who received a SMN diagnosis were at increased risk of suicide death. The risk elevation was most prominent soon after the cancer diagnosis, highlighting the necessity of providing timely psychological support to cancer survivors with a SMN.

## 1. Introduction

A growing number of patients are surviving from cancer because of the increased survival as well as the advances in cancer screening programs and novel treatments [1–3]. However, all cancer survivors are at risk for developing a second primary cancer [4–6]. It was reported that the incidence of second malignant neoplasm (SMN), calculated from the Surveillance, Epidemiology, and End Results (SEER) Program, was approximately 8.1% between 1992 and 2008 in the United States, indicating that nearly 1 in 12 cancer patients can develop a SMN [6].

Cancer is a devastating illness that comes with tremendous psychological distress [7–9]. Accumulating evidence has shown that people diagnosed with cancer are at an elevated risk of suicidal behaviors, including complete suicide [10–12] and suicide attempts [13, 14], compared with the general population. Therefore, it is plausible that receiving a diagnosis of SMN, acting as a repeat and possibly stronger stressor, might further increase the risk of suicide among cancer survivors. Indeed, patients with subsequent primary cancers (i.e., two or more primary cancers) have been reported to be at elevated risk for poorer physical and mental health status (e.g., serious psychological distress) [15–18]. However, the risk of suicide among patients with SMN is unclear.

Therefore, leveraging the population-based cancer cohort from the SEER database, which has stringent criteria for identifying multiple primary neoplasms, together with the aggregated US population data and US mortality data, we aimed to examine the risk of suicide among cancer survivors who developed a SMN.

## 2. Materials and Methods

### 2.1. Study Population

The SEER Program has collected cancer incidence and survival data from population-based cancer registries since 1973, covering approximately 34% of the US population as of 2016 [19]. Information on patient demographics, year and month of diagnosis, tumor characteristics (including primary tumor site, cancer behavior, tumor size, and tumor grade), treatment utilization, and active follow-up for vital status were routinely documented [20].

**Figure 1:**
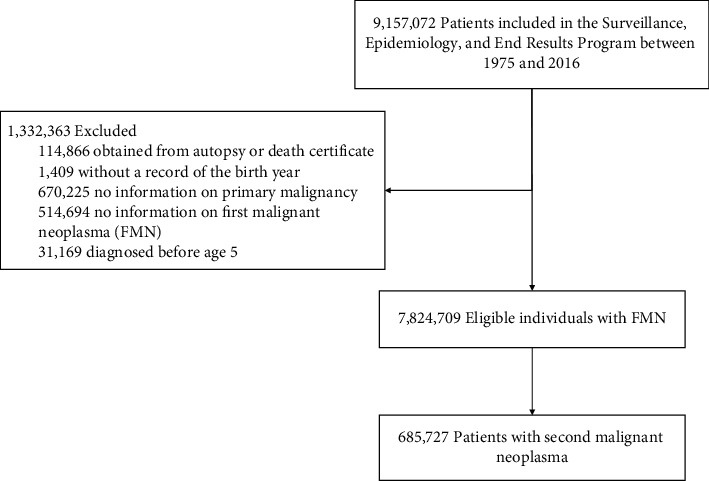
Study design.

In the present study, we conducted a population-based cohort study using the SEER registry records between January 1, 1975, and December 31, 2016, in the United States. Among the 9,157,072 cancer patients (Figure 1), we excluded 114,866 who were diagnosed only through autopsy or death certificate, 1,409 with no information on birth year, 1,184,919 that recorded as not their first malignancy (670,225 with no information on primary malignancy and 514,694 with no information on FMN), and 31,169 with their cancer diagnosis before age 5, leaving 7,824,709 eligible FMN patients for further analysis.

All patients were followed from the diagnosis of FMN until death, the occurrence of a subsequent malignancy, or December 31, 2016, whichever occurred first. Because the SEER data provided only survival in months for survival analysis, we assigned an average survival time of 15 days for individuals who died in the same month of diagnosis. By linking to the subsequent malignancy diagnoses in SEER, we identified 685,727 patients who developed a SMN during the follow-up who therefore were included in the exposed group. Namely, patients without a SMN diagnosis contributed all person-time to the unexposed group, while the ones with a SMN diagnosis contributed their person-time before the SMN diagnosis to the unexposed group and to the exposed group from the time of diagnosis onward.

### 2.2. Ascertainment of FMN and SMN

We first identified patients with a FMN diagnosis according to the SEER collected tumor information on the primary tumor (yes or no), tumor behavior (malignant or not), and first malignant primary indicator (yes or no). Through linkage to the information on subsequent cancer diagnoses, we then further identified individuals with a SMN diagnosis among these FMN survivors. In a subanalysis, we performed separate analyses on prostate cancer, breast cancer, colorectal cancer, lung cancer, nonmelanoma skin cancer, cancer of the central nervous system, severe cancer (esophageal, liver, or pancreatic cancer), and other cancers, as well as lifestyle-related cancers (alcohol- and smoking-related cancers) [21], according to the International Classification of Disease Oncology (ICD-O) third edition codes (Supplementary Table 1).

### 2.3. Ascertainment of Suicide

Based on the cause-specific death classification both for SEER and US mortality data, which was derived from the death certificate, we considered individuals to have committed suicide if the cause of death was coded as suicide and self-inflicted injury, with the corresponding International Classification of Diseases (ICD) 10th edition (ICD-10) codes as U03, X60-X84, and Y87.0.

### 2.4. Covariates

Data on potential confounders, including birth year, sex, calendar year at diagnosis, sociodemographic factors (i.e., race, cohabitation status, insurance, and state), tumor characteristic (i.e., tumor size and grade), and cancer treatment (i.e., chemotherapy, radiotherapy, and surgery), were obtained from the SEER database. All missing values of the covariates were coded to the “unknown” category. The corresponding information on age at death, sex, and calendar year for the US population data were derived from the US Census Bureau's Population Estimates Program.

### 2.5. Statistical Analysis

We assessed the relative risk of suicide in relation to SMN diagnosis compared with FMN patients, using hazard ratios (HRs) with 95% confidence intervals (CI) derived from Cox regression models. Models were partly (models 1–3) or fully (model 4) adjusted for the birth year (as a continuous variable), sex (male or female), calendar year at diagnosis (1975–1984, 1985–1994, 1995–2004, or 2005–2016), race (white, black, or others/unknown), cohabitation status (cohabitation, noncohabitation, or unknown), insurance (insured, uninsured, or unknown), state (Alaska, California, Connecticut, Georgia, Hawaii, Iowa, Kentucky, Louisiana, Michigan, New Jersey, New Mexico, Utah, or Washington), tumor size (<1.5, ≥1.5 cm, or unknown), tumor grade (well, moderately, poorly differentiated/undifferentiated, or unknown), chemotherapy/radiotherapy (yes or no/unknown), and surgery (yes or no/unknown). We also examined the association between SMN diagnosis and subsequent risk of suicide death by cancer subtypes, after controlling for available confounders. Lifestyle factors (i.e., alcohol drinking and smoking) might modify the studied association. We therefore also separately analyzed alcohol-related cancers and smoking-related cancers.

In subgroup analyses, HRs were calculated separately by age at diagnosis (by quantile: <55, 55–65, 66–75, or >75 years), calendar year at diagnosis, sex, race, cohabitation status, tumor size, tumor grade, chemotherapy/radiotherapy, surgery, and the time interval between FMN and SMN. Furthermore, we explored whether the relative risk of suicide after a SMN diagnosis varied across different time periods since cancer diagnosis, by analyzing the associations in different follow-up periods (<1, 1–2, 3–4, 5–10, or >10 years). Additionally, we calculated the HRs by characteristics of their first malignancies (i.e., tumor characteristics and treatment utilization) to explore the potential modified role of first malignancy among SMN patients. The statistical significance of the difference between HRs was assessed by including an interaction term in the Cox model.

To better interpret our findings, we also calculated standardized mortality ratios (SMRs; i.e., the ratio of the observed to the expected number of suicide deaths) with 95% CIs to estimate the relative risk for suicide after SMN, as well as FMN, using the general US population as reference. Specifically, the number of expected suicide deaths was calculated by multiplying the observed number of person-years by age- (5 year strata), sex-, and calendar-year-specific suicide rates derived from the general US population. We further estimated SMRs by different follow-up periods (i.e., ≤1, 2–3, 4–6, or 7–12 months or 1–2, 3–5, 6–10, or >10 years) after the SMN or FMN diagnosis to examine the immediate and long-term impact. The statistical significance of the difference between SMRs was assessed by the heterogeneity test.

To test the robustness of the observed associations, we repeated the main analysis excluding SMN patients with a prior history of FMN at the same tumor site. We further assessed the potential influence of unmeasured confounders using the *E*-value [22]. All the analyses were conducted in R software (version 4.0). A two-sided *P*-value <0.05 was considered statistically significant.

## 3. Results

Of the 9,157,072 cancer patients in the SEER data, we identified 7,824,709 FMN patients in the population-based cohort, among which 685,727 individuals were exposed to a SMN diagnosis during up to 42 years of the follow-up period (Figure 1). With a total of 46,507,112 accumulated person-years, the median follow-up time was 1.67 (Q1–Q3: 0.42–5.00) and 3.17 (0.67–8.67) years for the SMN and FMN patients, respectively (Table 1). There was little difference in race, cohabitation status, state, and tumor grade between the SMN and FMN patients. However, compared with the FMN patients, patients with a SMN diagnosis were more likely to be male (58.97% vs. 51.28%), older (mean age 71.00 vs. 63.60 years), insured (49.46% vs. 38.82%), with larger tumor size (46.92% vs. 42.31%), and not treated (37.35% vs. 44.32% and 53.46% vs. 58.59% for chemotherapy/radiotherapy and surgery, respectively).

**Figure 2:**
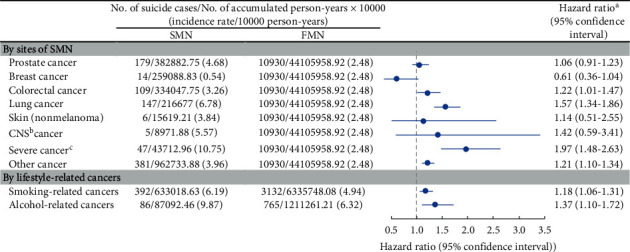
Risk of suicide among individuals with a second malignant neoplasm (SMN) diagnosis, by cites of SMN or lifestyle-related cancers, compared with individuals with a first malignant neoplasm (FMN) diagnosis. ^a^Cox model was used to estimate hazard ratios (HRs), adjusted for birth year, sex, calendar year, race, cohabitation status, insurance, state, tumor size, grade, chemotherapy/radiotherapy, and surgery. ^b^Central nervous system. ^c^Esophageal, liver, or pancreatic cancer.

During follow-up, a total of 937 suicide deaths (crude incidence rate, 3.90 per 10,000 person-years) and 10,930 suicide deaths (2.48 per 10,000 person-years) were identified among SMN patients and FMN patients, respectively (Table 2). Correspondingly, we observed a consistently elevated relative risk of suicide among SMN patients compared with FMN patients across different models; the fully adjusted HR (model 4) was 1.23 (95% CI 1.14–1.31). Similar risk elevations were observed for alcohol-related cancers (HR 1.37; 95% CI 1.10–1.72; Figure 2) and smoking-related cancers (HR 1.18; 95% CI 1.06–1.31). Furthermore, among all studied cancer subtypes defined by the sites of SMN, the HRs were most pronounced when SMN was severe cancer (HR 1.97; 95% CI 1.48–2.63) or lung cancer (HR 1.57; 95% CI 1.34–1.86; Figure 2).

In a subanalysis, the excess risk of suicide in SMN patients did not differ by calendar year, sex, race, cohabitation status, tumor grade, tumor size, chemotherapy/radiotherapy, and the time interval between FMN and SMN (Table 3). Likewise, similar estimates were found across different follow-up periods (*P*_difference_ = 0.95; Table 3). However, the HRs were somewhat higher among younger patients (*P*_difference_ = 0.0003) and patients treated with surgery (*P*_difference_ = 0.0011). A greater risk of suicide was also observed in SMN patients who underwent chemotherapy/radiotherapy (*P*_difference_ = 0.013) and surgery (*P*_difference_ = 0.045) for their first malignancy (Table 4).

**Figure 3:**
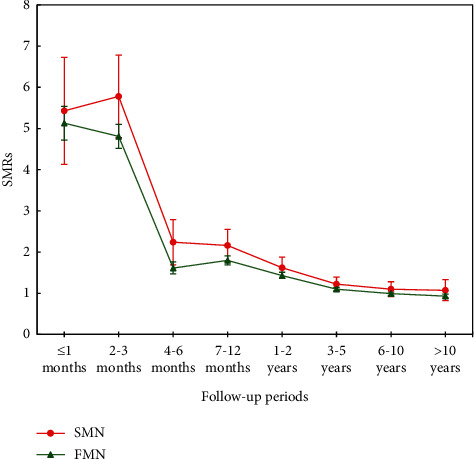
Change of the suicide standardized mortality ratios (SMRs) over follow-up periods among patients with first malignant neoplasm (FMN) or second malignant neoplasm (SMN), compared with the general population. The expected number for suicide deaths during the study period was derived from age (5 year groups), sex, and calendar year (1 year groups) suicide death rate for the USA.

The calculation of SMRs, using the general US population as a reference, revealed a further increased risk of suicide among SMN patients (SMR 1.65 (95% CI 1.54–1.75)) relative to FMN patients (SMR 1.29 (95% CI 1.26–1.31); *P*_difference_ < 0.0001). Across different follow-up periods, the changing patterns were similar between SMN patients and FMN patients, both of which showed the highest estimates within the first 3 months after cancer diagnosis (Figure 3 and Supplementary Table 2). The excess risk then experienced a rapid decline but remained at a significant level for up to 5 years.

In sensitivity analysis, we observed largely comparable results after excluding SMN patients whose first malignancy was at the same tumor site (Supplementary Table 3). In addition, the calculation of the *E*-value revealed that a minimal magnitude of 1.76-fold increased risk of suicide death that was associated with the unmeasured confounders would be needed to entirely explain the observed association.

## 4. Discussion

To the best of our knowledge, this is the first large-scale cohort study that examined the risk of suicide among cancer survivors who developed a SMN, using population-based register data in the USA. We found that compared with FMN patients, patients with a SMN diagnosis, particularly those diagnosed at a younger age, with aggressive SMN, or had received surgery treatment for the SMN, were at an increased risk of suicide death within the entire follow-up period after adjusting for many potential confounders. Importantly, as the calculations of relative risk (i.e., SMR) of suicidal death relative to the general US population revealed a high-risk time period (i.e., the first 3 months after SMN diagnosis), such finding highlights the time window for suicide intervention among SMN patients.

The immediate suicide risk following a FMN diagnosis has been well estimated in previous studies, suggesting cancer diagnosis as an acute stressor that can lead to suicide behaviors [11, 13, 23]. Nevertheless, with regard to SMN, data from large longitudinal studies are scarce. We therefore have limited knowledge about the suicide risk among SMN patients, and whether the level of such a risk is comparable or superior to that among FMN patients. In our study, we, for the first time, showed a significant impact of a SMN diagnosis on the subsequent suicide risk, relative to a FMN diagnosis. Despite the absence of comparable data from studies with a similar design, our findings gain support from previous investigations that consistently suggested cancer survivors with multiple primary cancers had more frequent or greater psychological distress than survivors of single cancer, which was measured by the number of days of self-reported mental-health-related feelings in the past month [15–17]. Such results indicate that a diagnosis of subsequent primary cancer might bring additional adverse effects on the mental health among cancer survivors. Moreover, in a study also based on the SEER database, it focused on the sex disparity of suicide risk among cancer patients, and the authors reported an increased risk for suicidal death among secondary primary head and neck cancer patients, compared with those with head and neck cancer as the first primary cancer, which is in line with our present results [24]. Furthermore, the comparison with the general population corroborates the findings by illustrating consistently higher SMRs among SMN than FMN patients over time since the diagnosis. Those two groups of patients, however, shared a temporal pattern of suicide death risk, with the period shortly after the cancer diagnosis (i.e., within 3 months for both SMN and FMN) as a high-risk time period with the most frequent suicide deaths. The observed temporal pattern was in accordance with our previous findings on the risk patterns of mental disorders after the cancer diagnosis [8]. These results further indicate the psychological stress induced by the diagnosis of the subsequent malignant neoplasm and call for attention to monitor the mental health status of cancer survivors, especially for those who experienced a recent diagnosis of cancer, and to carry out necessary thought interventions, such as initiating timely psychosocial care [25] and providing prompt suicide risk screening [26].

Besides the repeated exposure to a notorious stressful event such as cancer diagnosis [27], other explanations for the phenomenon that SMN patients might suffer from a higher level of psychological stress, in comparison with FMN patients, include poorer mental health status (e.g., being more likely to have the serious psychological disorder) [15], worse overall health condition (e.g., being reported more health-related bed days) [15], poorer quality of life [28], and undermined socioeconomic status due to the treatment of FMN [29]. In addition, even if patients eventually survived their cancer, cancer treatment itself could be invasive and painful and often accompanied by severe side effects that could traumatize cancer survivors for an extended period of time [30]. Therefore, the fear of cancer treatment can be another underlying trigger that contributes to more suicidal behaviors among SMN patients. Indeed, in our analysis, we found SMN patients who underwent chemotherapy/radiotherapy or surgery for their FMN had a higher relative risk for suicide death, providing supportive evidence to this notion.

The major merits of our study include the large population-based sample of cancer survivors across the USA and the complete follow-up period, which ensures the presentiveness of the study sample. Also, the large sample size enabled detailed analyses for all subgroups, especially by different follow-up times since diagnosis. The data on cancer diagnosis and cause of death are collected prospectively and independently, which minimizes the risk of information bias. In addition, using the enriched information on tumor characteristics and cancer treatment, we were able to control for important tumor- and treatment-related confounders in our analysis.

Our study has several limitations. First, the misclassification of some causes of death may be a concern. However, it is unlikely that the misclassification would differ substantially between patients with malignancy and the general population. Second, the SEER program merely recruited cancer patients, lacking a cancer-free group as a reference. However, the comparisons of the suicide rates between our study population and the age-, calendar-year-, and sex-matched general population through SMR calculation facilitate the identification of high-risk time window of suicide for timely and effective intervention. Third, due to the lack of information on some potential confounders, such as lifestyle factors, preexisting psychiatric disorders, and history of somatic illness, the residual confounding could be an issue. During analysis, we have made extra efforts by analyzing lifestyle-related cancers (i.e., alcohol- and smoking-related cancers) separately. As such analyses led to comparable estimates, it seems the lifestyle factor did not substantially modify the observed associations. The role of preexisting psychiatric disorders and history of somatic illness need to be assessed in further investigations. However, the calculated *E*-value suggested that the observed association was unlikely to be entirely explained by the unmeasured confounding. Finally, the generalization of our findings to the entire nation or other populations needs cautions since SEER areas had better case completeness, greater economic disadvantage, and greater minority diversity than non-SEER areas [31]. Despite these discrepancies, we assume these factors should have a limited effect on the studied association as we compared individuals with FMN to those with SMN in our study, all of which come from SEER areas.

In conclusion, this population-based cohort in the USA indicated that cancer survivors receiving a SMN diagnosis were at an increased risk of suicide, compared with either patients with FMN or the general population. The excess risk was most pronounced within the time period immediately after the SMN diagnosis, highlighting the necessity of providing timely psychological support to patients suffering SMN.

## Figures and Tables

**Table 1:** Characteristics of the study cohort.

	SMN^a^(*N* = 685727)	FMN^b^(*N* = 7824709)
Follow-up time, mean (SD), y	3.50 (4.47)	5.64 (6.53)
Follow-up time, median (Q1–Q3), y	1.67 (0.42–5.00)	3.17 (0.67–8.67)
Birth year, mean (SD), y	1,930 (14.70)	1,940 (18.50)
Age at diagnosis, mean (SD), y	71.00 (11.60)	63.60 (15.10)

Age group (by quantile), no. (%), y		
<55	58,180 (8.48)	1,939,983 (24.79)
55–66	159,108 (23.20)	2,300,439 (29.40)
67–75	206,746 (30.15)	1,827,392 (23.35)
>75	261,693 (38.16)	1,756,895 (22.45)

Calendar year, no. (%)		
1975–1984	26,808 (3.91)	668,993 (8.55)
1985–1994	74,360 (10.84)	988,075 (12.63)
1995–2004	163,161 (23.79)	2,194,895 (28.05)
2005–2016	421,398 (61.45)	3,972,746 (50.77)

Sex, no. (%)		
Male	404,391 (58.97)	4,012,867 (51.28)
Female	281,336 (41.03)	3,811,842 (48.72)

Race, no. (%)		
White	586,494 (85.53)	6,427,454 (82.14)
Black	63,114 (9.20)	797,649 (10.19)
Others/unknown	36,119 (5.27)	599,606 (7.66)

Cohabitation status, no. (%)		
Cohabitation	249,999 (36.46)	2,940,863 (37.58)
Noncohabitation	390,221 (56.91)	4,351,566 (55.61)
Unknown	45,507 (6.64)	532,280 (6.80)

Insurance, no. (%)		
Insured	339,146 (49.46)	3,037,767 (38.82)
Uninsured	3,822 (0.56)	90,885 (1.16)
Unknown	342,759 (49.98)	4,696,057 (60.02)

State, no. (%)		
Alaska	533 (0.08)	7,495 (0.10)
California	216,848 (31.62)	2,767,483 (35.37)
Connecticut	63,227 (9.22)	603,280 (7.71)
Georgia	56,227 (8.20)	733,285 (9.37)
Hawaii	15,738 (2.30)	169,385 (2.16)
Iowa	55,057 (8.03)	508,535 (6.50)
Kentucky	26,320 (3.84)	337,028 (4.31)
Louisiana	23,977 (3.50)	320,179 (4.09)
Michigan	73,514 (10.72)	676,773 (8.65)
New Jersey	52,786 (7.70)	664,090 (8.49)
New Mexico	17,921 (2.61)	223,539 (2.86)
Utah	19,001 (2.77)	220,849 (2.82)
Washington	64,578 (9.42)	592,788 (7.58)

Cancer sites, no. (%)		
Prostate cancer	70,992 (10.35)	1,186,195 (15.16)
Breast cancer	45,939 (6.70)	1,188,792 (15.19)
Colorectal cancer	82,991 (12.10)	835,070 (10.67)
Lung cancer	121,001 (17.65)	975,192 (12.46)
Skin (nonmelanoma)	4,064 (0.59)	28,036 (0.36)
CNS^c^ cancer	6,630 (0.97)	113,471 (1.45)
Severe cancer^d^	40,647 (5.93)	388,774 (4.97)
Other cancers	258,521 (37.70)	2,526,393 (32.29)

Smoking-related cancers		
No	434,306 (63.34)	5,814,303 (74.31)
Yes	251,421 (36.66)	2,010,406 (25.69)

Alcohol-related cancers		
No	648,254 (94.54)	7,467,584 (95.44)
Yes	37,473 (5.46)	357,125 (4.56)

Tumor size, no. (%), cm		
<1.5	66,741 (9.73)	791,353 (10.11)
≥1.5	321,740 (46.92)	3,310,859 (42.31)
Unknown	297,246 (43.35)	3,722,497 (47.57)

Tumor grade, no. (%)		
Well differentiated	61,799 (9.01)	734,335 (9.38)
Moderately differentiated	170,021 (24.79)	2,069,948 (26.45)
Poorly differentiated/undifferentiated	155,702 (22.71)	1,900,555 (24.29)
Unknown	298,205 (43.49)	3,119,871 (39.87)

Chemotherapy/radiotherapy		
No/unknown	429,626 (62.65)	4,356,691 (55.68)
Yes	256,101 (37.35)	3,468,018 (44.32)

Surgery		
No/unknown	319,150 (46.54)	3,240,379 (41.41)
Yes	366,577 (53.46)	4,584,330 (58.59)

^a^Second malignant neoplasm. ^b^First malignant neoplasm. ^c^Central nervous system. ^d^Esophageal, liver, or pancreatic cancer.

**Table 2:** Risk of suicide among individuals with a second malignant neoplasm (SMN) diagnosis, by different adjustment strategies, compared with individuals with a first malignant neoplasm (FMN) diagnosis.

Model information	No. of suicide cases/no. of accumulated person-years × 10,000 (incidence rate/10,000 person-years)		Hazard ratio (95% confidence interval)
	SMN	FMN	
Different adjustment strategies^a^			
Model 1: adjusted for birth year, sex, and calendar year	937/2,401,152.92 (3.90)	10930/44,105,958.92 (2.48)	1.24 (1.16–1.32)
Model 2: model 1 + sociodemographic factors (i.e., race, cohabitation status, insurance, and state)			1.23 (1.15–1.32)
Model 3: model 2 + tumor characteristics (i.e., tumor size and tumor grade)			1.22 (1.14–1.31)
Model 4 (full model): model 3 + cancer treatment (i.e., chemotherapy/radiotherapy and surgery)			1.23 (1.14–1.31)

^a^Cox model was used to estimate hazard ratios (HRs), adjusted for covariates listed in the model information column.

**Table 3:** Risk of suicide among individuals with a second malignant neoplasm (SMN) diagnosis, by different characteristics, compared with individuals with a first malignant neoplasm (FMN) diagnosis.

	No. of suicide cases/no. of accumulated person-years × 10,000 (incidence rate/10,000 person-years)		Hazard ratio^a^ (95% confidence interval)	*P*_difference_
	SMN	FMN		
By age at diagnosis (by quantile), y				0.0003
<55	75/315,337.92 (2.38)	2780/15,810,144.58 (1.76)	1.38 (1.09–1.73)	
55–65	223/687,006.17 (3.25)	3234/13,794,478.21 (2.34)	1.32 (1.15–1.52)	
66–75	339/747,527.17 (4.53)	2816/9,059,181.29 (3.11)	1.33 (1.19–1.50)	
>75	300/651,281.67 (4.61)	2100/5,442,154.83 (3.86)	0.96 (0.85–1.09)	
By calendar year, y				0.15
1975–1984	68/116,017.71 (5.86)	1584/5,375,711.83 (2.95)	1.43 (1.12–1.82)	
1985–1994	191/364,410.29 (5.24)	2296/8,174,559.13 (2.81)	1.40 (1.21–1.63)	
1995–2004	260/789,908.79 (3.29)	3473/16,284,736.13 (2.13)	1.21 (1.06–1.37)	
2005–2016	418/1,130,816.13 (3.70)	3577/14,270,951.83 (2.51)	1.17 (1.05–1.29)	
By sex				0.99
Male	825/1,320,654.54 (6.25)	9030/20,956,633.67 (4.31)	1.20 (1.11–1.29)	
Female	112/1,080,498.38 (1.04)	1900/23,149,325.25 (0.82)	1.20 (0.99–1.46)	
By race				0.066
White	880/2,091,732.79 (4.21)	10060/36,981,749.92 (2.72)	1.23 (1.15–1.32)	
Black	38/181,568.13 (2.09)	368/3,798,398.25 (0.97)	1.70 (1.21–2.41)	
By cohabitation status				0.45
Cohabitation	336/746,244.92 (4.50)	4120/13,911,216.5 (2.96)	1.20 (1.07–1.35)	
Noncohabitation	546/1,493,855.42 (3.65)	5939/27,156,872.79 (2.19)	1.27 (1.16–1.39)	
By tumor size, cm				0.057
<1.5	79/321,027 (2.46)	758/5,869,076.38 (1.29)	1.39 (1.10–1.76)	
≥1.5	366/1,025,931.79 (3.57)	3640/16,163,041.38 (2.25)	1.08 (0.97–1.21)	
By tumor grade				0.81
Well differentiated	86/306,398.13 (2.81)	1000/5,384,767.58 (1.86)	1.29 (1.03–1.61)	
Moderately differentiated	275/769,611.54 (3.57)	3179/14,044,744.63 (2.26)	1.37 (1.21–1.56)	
Poorly differentiated/undifferentiated	252/473,608.54 (5.32)	2696/8,978,857.25 (3.00)	1.40 (1.23–1.60)	
By chemotherapy/radiotherapy				0.91
No/unknown	630/1,590,770.13 (3.96)	6701/25,942,336.79 (2.58)	1.22 (1.13–1.33)	
Yes	307/810,382.79 (3.79)	4229/18,163,622.13 (2.33)	1.23 (1.09–1.38)	
By surgery				0.0011
No/unknown	397/662,482.42 (5.99)	4654/10,600,572.79 (4.39)	1.07 (0.96–1.18)	
Yes	540/1,738,670.5 (3.11)	6276/33,505,386.13 (1.87)	1.35 (1.23–1.48)	
By time interval between FMN and SMN, y				0.84
≤1	246/630,900.92 (3.90)	10930/44,105,958.92 (2.48)	1.24 (1.09–1.40)	
2–3	195/464,630.75 (4.20)	10930/44,105,958.92 (2.48)	1.29 (1.12–1.49)	
4–6	189/486,033.92 (3.89)	10930/44,105,958.92 (2.48)	1.19 (1.03–1.38)	
>6	307/819,587.33 (3.75)	10930/44,105,958.92 (2.48)	1.20 (1.07–1.34)	
By follow-up periods, y				0.95
<1	376/509,112.92 (7.39)	3131/6,261,779.63 (5.00)	1.18 (1.06–1.31)	
1–2	153/784,901.33 (1.95)	1510/10,511,420.75 (1.44)	1.26 (1.06–1.49)	
3–4	199/1,328,072.67 (1.50)	2368/20,421,357.75 (1.16)	1.22 (1.06–1.42)	
5–10	143/1,387,784.33 (1.03)	2210/26,923,811.17 (0.82)	1.24 (1.05–1.48)	
>10	66/904,527.5 (0.73)	1711/26,133,082.67 (0.65)	1.28 (1.00–1.63)	

^a^Cox model was used to estimate hazard ratios (HRs), adjusted for birth year, sex, calendar year, race, cohabitation status, insurance, state, tumor size, grade, chemotherapy/radiotherapy, and surgery.

**Table 4:** Risk of suicide among individuals with a second malignant neoplasm (SMN) diagnosis, by characteristics of the first malignancy, compared with individuals with a first malignant neoplasm (FMN) diagnosis.

	No. of suicide cases/no. of accumulated person-years × 10,000 (incidence rate/10,000 person-years)		Hazard ratio^a^ (95% confidence interval)	*P*_difference_
	SMN	FMN		
By FMN tumor size, cm				0.57
<1.5	62/260,141.08 (2.38)	758/5,869,076.38 (1.29)	1.55 (0.77–3.12)	
≥1.5	285/839,718.17 (3.39)	3,640/16,163,041.38 (2.25)	1.26 (1.06–1.50)	
By FMN grade				0.54
Well differentiated	118/318,247.38 (3.71)	1,000/5,384,767.58 (1.86)	1.39 (0.82–2.36)	
Moderately differentiated	334/817,983.33 (4.08)	3,179/14,044,744.63 (2.26)	1.65 (1.37–1.99)	
Poorly differentiated/undifferentiated	212/466,502.54 (4.54)	2,696/8,978,857.25 (3.00)	1.86 (1.47–2.37)	
By FMN chemotherapy/radiotherapy				0.013
No/unknown	590/1,525,397.25 (3.87)	6,701/25,942,336.79 (2.58)	1.28 (1.16–1.41)	
Yes	347/875,755.67 (3.96)	4,229/18,163,622.13 (2.33)	1.64 (1.39–1.95)	
By FMN surgery				0.045
No/unknown	272/512,397.08 (5.31)	4,654/10,600,572.79 (4.39)	1.18 (0.99–1.40)	
Yes	665/1,888,755.83 (3.52)	6,276/33,505,386.13 (1.87)	1.45 (1.31–1.61)	

^a^Cox model was used to estimate hazard ratios (HRs), adjusted for birth year, sex, calendar year, race, cohabitation status, insurance, state, tumor size, grade, chemotherapy/radiotherapy, and surgery.

## Data Availability

The data analyzed in this study were obtained from the Surveillance, Epidemiology, and End Results Program (https://seer.cancer.gov/).

## Supplementary Materials

Supplementary Materials Supplementary Table 1: International Classification of Disease Oncology (ICD-O) third version codes for diagnoses used in this study. Supplementary Table 2: change of the suicide standardized mortality ratios (SMRs) over follow-up periods among patients with first malignant neoplasm (FMN) or second malignant neoplasm (SMN), compared with the general population. Supplementary Table 3: sensitive analysis of the risk of suicide among individuals with a second malignant neoplasm (SMN) diagnosis, by excluding SMN patients with a prior history of first malignant neoplasm (FMN) at the same tumor site (*N* = 40,076), compared with individuals with FMN.
