# Supplementary material for: Increased Risk of Suicide among Cancer Survivors Who Developed a Second Malignant Neoplasm
Source: Comput Intell Neurosci. 2022 Jan 10;2022:2066133. doi: 10.1155/2022/2066133 (PMC8763535; doi:10.1155/2022/2066133)
Supplement: Supplementary Materials — Supplementary Table 1: International Classification of Disease Oncology (ICD-O) third version codes for diagnoses used in this study. Supplementary Table 2: change of the suicide standardized mortality ratios (SMRs) over follow-up periods among patients with first malignant neoplasm (FMN) or second malignant neoplasm (SMN), compared with the general population. Supplementary Table 3: sensitive analysis of the risk of suicide among individuals with a second malignant neoplasm (SMN) diagnosis, by excluding SMN patients with a prior history of first malignant neoplasm (FMN) at the same tumor site (N = 40,076), compared with individuals with FMN. [file 2066133.f1.docx]

**Supplementary Table 1** **International Classification of Disease Oncology (ICD-O) third version codes for diagnoses used in this study**

|  | **ICD-O-3 codes** |
| --- | --- |
| **Any cancer** |  |
| Prostate cancer | C619 |
| Breast cancer | C500-C509 |
| Colorectal cancer | C180-C189, C199, C209-C212, C218, C260 |
| Lung cancer | C340-C349 |
| Non-melanoma skin cancer | C440-C449 |
| Cancer of the central nervous system | C700-C729 |
| Lymphatic or hematopoietic cancer | C024, C098-C099, C111, C142, C379, C420-C422, C424, C770-C779 |
| Severe cancer | C150-C159, C220-C221, C250-C259 |
| **Lifestyle-related cancers** |  |
| Alcohol-related cancers | C019-C029, C030-C069, C100-C119, C140, C142, C148, C150-C159, C220, C320-C329 |
| Smoking-related cancers | C000-C009, C019-C069, C100-C119, C129-C140, C142, C148, C150-C159, C250-C259, C300-C301, C310-C329, C340-C349, C649, C659, C669-C679, C680-C689 |

**Supplementary Table 2 Change of the suicide standardized mortality ratios (SMRs) over follow-up periods among patients with first malignant neoplasm (FMN) or second malignant neoplasm (SMN), compared with the general population**

| **Follow-up periods** | **SMR (95% confidence interval)**^a^ | |
| --- | --- | --- |
|  | **SMN** | **FMN** |
| **Overall** | **1.65 (1.54-1.75)** | **1.29 (1.26-1.31)** |
| ≤1 months | 5.43 (4.13-6.73) | 5.13 (4.72-5.54) |
| 2-3 months | 5.78 (4.78-6.78) | 4.81 (4.52-5.10) |
| 4-6 months | 2.24 (1.69-2.79) | 1.61 (1.46-1.75) |
| 7-12 months | 2.16 (1.77-2.55) | 1.80 (1.69-1.91) |
| 1-2 years | 1.62 (1.36-1.88) | 1.43 (1.35-1.50) |
| 3-5 years | 1.22 (1.05-1.38) | 1.10 (1.06-1.15) |
| 6-10 years | 1.10 (0.92-1.28) | 0.99 (0.95-1.03) |
| >10 years | 1.07 (0.81-1.32) | 0.93 (0.88-0.97) |

^a^ The expected number for suicide deaths during the study period was derived from age (5 year groups), sex, and calendar-year (1 year groups) suicide death rate for the USA.

**Supplementary Table 3 Sensitive analysis of risk of suicide among individuals with a second malignant neoplasm (SMN) diagnosis, by excluding SMN patients with a prior history of first malignant neoplasm (FMN) at the same tumor site (N=40,076), compared with individuals with FMN**

|  | **No. of suicide cases/No. of accumulated person-years × 10000 (incidence rate/10000 person-years)** | | **Hazard Ratio^a^**  **(95% confidence interval)** |
| --- | --- | --- | --- |
|  | **SMN** | **FMN** |  |
| **Full model** | 925/2401152.92 (3.85) | 10908/44105958.92 (2.47) | 1.23 (1.15-1.31) |
| **By sites of SMN** |  |  |  |
| Prostate cancer | 179/382882.75 (4.68) | 10908/44105958.92 (2.47) | 1.06 (0.91-1.23) |
| Breast cancer | 14/259088.83 (0.54) | 10908/44105958.92 (2.47) | 0.62 (0.37-1.05) |
| Colorectal cancer | 109/334047.75 (3.26) | 10908/44105958.92 (2.47) | 1.22 (1.01-1.47) |
| Lung cancer | 139/216677 (6.42) | 10908/44105958.92 (2.47) | 1.61 (1.36-1.91) |
| Skin (nonmelanoma) | 6/15619.21 (3.84) | 10908/44105958.92 (2.47) | 1.15 (0.52-2.57) |
| CNS^b^ cancer | 5/8971.88 (5.57) | 10908/44105958.92 (2.47) | 1.42 (0.59-3.42) |
| Severe cancer^c^ | 47/43712.96 (10.75) | 10908/44105958.92 (2.47) | 1.98 (1.48-2.63) |
| Other cancer | 426/1140152.54 (3.74) | 10908/44105958.92 (2.47) | 1.21 (1.10-1.34) |

^a^ Cox model was used to estimate hazard ratios (HRs), adjusted for birth year, sex, calendar year, race, cohabitation status, insurance, state, tumor size, grade, chemotherapy/radiotherapy, and surgery.

^b^ Central nervous system

^c^ Esophageal, liver, or pancreatic cancer
